# The Peptidyl Prolyl Isomerase Rrd1 Regulates the Elongation of RNA Polymerase II during Transcriptional Stresses

**DOI:** 10.1371/journal.pone.0023159

**Published:** 2011-08-24

**Authors:** Jeremie Poschmann, Simon Drouin, Pierre-Etienne Jacques, Karima El Fadili, Michael Newmarch, François Robert, Dindial Ramotar

**Affiliations:** 1 Department of Medicine, Maisonneuve-Rosemont Hospital, Research Center, University of Montreal, Montréal, Québec, Canada; 2 Institut de recherches cliniques de Montréal, Montréal, Québec, Canada; 3 Département de Médecine, Faculté de Médecine, Université de Montréal, Montréal, Québec, Canada; George Mason University, United States of America

## Abstract

Rapamycin is an anticancer agent and immunosuppressant that acts by inhibiting the TOR signaling pathway. In yeast, rapamycin mediates a profound transcriptional response for which the *RRD1* gene is required. To further investigate this connection, we performed genome-wide location analysis of RNA polymerase II (RNAPII) and Rrd1 in response to rapamycin and found that Rrd1 colocalizes with RNAPII on actively transcribed genes and that both are recruited to rapamycin responsive genes. Strikingly, when Rrd1 is lacking, RNAPII remains inappropriately associated to ribosomal genes and fails to be recruited to rapamycin responsive genes. This occurs independently of TATA box binding protein recruitment but involves the modulation of the phosphorylation status of RNAPII CTD by Rrd1. Further, we demonstrate that Rrd1 is also involved in various other transcriptional stress responses besides rapamycin. We propose that Rrd1 is a novel transcription elongation factor that fine-tunes the transcriptional stress response of RNAPII.

## Introduction

Rapamycin is an immunosuppressant and an anticancer molecule that acts through inhibition of the TOR (target of rapamycin) signaling pathway [1]. In the yeast *Saccharomyces cerevisiae*, *TOR1* and *TOR2* encode serine/threonine kinases that form the core of the rapamycin sensitive (TORC1) and the rapamycin insensitive (TORC2) complex, respectively [2], [3]. TORC1 positively regulates anabolic processes by promoting mRNA translation and the transcription of ribosome biogenesis genes [2], [3]. Upon nutrient starvation or rapamycin treatment, the TORC1 complex becomes inactivated, with the consequence of a severe reduction of anabolic processes, cell cycle progression and growth, as well as the induction of catabolic processes and stress responsive factors [2], [3]. These drastic changes are driven by important alterations of gene transcription mediated, at least in part, by the translocation of transcription factors to the nucleus. Upon TORC1 inhibition, the ribosomal gene repressor, Crf1, translocates into the nucleus to inhibit ribosomal gene transcription [4]. Furthermore, the TORC1 regulator Tap42 activates PP2A and Sit4 phosphatases, which in turn dephosphorylate the transcription factors Rtg1/2 and Gln3 causing them to move into the nucleus and induce the expression of retrograde signalling genes (RTG) and nitrogen discrimination genes (NDG), respectively [2], [5], [6], [7]. Once translocated into the nucleus, these transcription factors bind to specific DNA elements, alter the local chromatin state and recruit the general transcription machinery to mediate pre-initiation complex (PIC) assembly and transcription by RNAPII [8], [9].

The exact mechanisms of these regulatory circuits are not fully understood but genome wide deletion screens in *S.cerevisae* have been a useful tool to identify novel factors that are required to mediate an efficient response to rapamycin [10], [11], [12], [13]. One of these factors is the peptidyl prolyl isomerase Rrd1 (Resistant to rapamycin deletion 1). *Rrd1Δ* mutants exhibit multiple phenotypes including sensitivity to the carcinogen 4-nitroquinoline-1-oxide and UVA radiation, and, most prominently, extreme resistance to rapamycin [13]. Rrd1 is evolutionally conserved and shares 35% identity with its human homologue PTPA [14]. PTPA was first characterized to be an activator of the phospho-tyrosyl phosphatase activity of PP2A phosphatases *in vitro* [15], [16]. However, an *in vivo* role for this activity has not yet been described, and subsequent studies revealed that PTPA as well as Rrd1 are required for PP2A substrate specificity, complex formation and the reactivation of inactive PP2A complexes [17], [18]. Both were later found to possess intrinsic peptidyl prolyl isomerase activity on a specific PP2A peptide [14]. Consistent with this function, we and others found that Rrd1 interacts with the yeast PP2A-like phosphatase Sit4 [19]. Sit4 and Rrd1 form a ternary complex with the Tor signaling mediator Tap42 [19]. As mentioned above, upon TORC1 inactivation Tap42 dissociates from Sit4-Rrd1, which then dephosphorylates and activates the transcription factor Gln3 [6], [20]. However, we found that the Gln3 target gene *MEP2* was activated independently of Rrd1, suggesting that this latter factor has an additional role in the response to rapamycin [20]. Consistent with this, we found that Rrd1 exerts an effect at the transcriptional level: genes known to be upregulated (the diauxic shift genes *CPA2* and *PYC1*) and down-regulated (the ribosomal protein genes including *RPS26A*, *RPL30*, and *RPL9*) following rapamycin exposure showed an altered transcription pattern in *rrd1Δ* mutants [20]. Since ribosomal biogenesis results from the concerted action of all three RNA polymerases, which are controlled by a tight regulatory network, we expected that Rrd1 plays a broader role in transcription of these genes [21]. Indeed, we subsequently found that Rrd1 is associated with the chromatin and that it interacts with the major subunit of RNAPII [22]. Further, biochemical analysis revealed that Rrd1 is able to release RNAPII from the chromatin *in vivo* and *in vitro*, which we ascribed to the peptidyl prolyl isomerase activity acting on the C-terminal domain (CTD) of RNAPII [22]. This mechanism of RNAPII regulation resembles that of the peptidyl prolyl isomerase, Pin1, and its yeast homologue Ess1 which are also known to regulate transcription [23], [24], [25], [26], [27]. Both Pin1 and Ess1 are thought to isomerize the CTD of RNAPII and regulate elongation [26], [27]. In yeast, the CTD consists of 26 repeats of the YS_2_PTS_5_PS_7_ heptad sequence which are differentially phosphorylated on Ser2, Ser5 and Ser7 [28], [29], [30], [31]. These different phosphorylation patterns act as a recruitment platform for multiple factors involved in chromatin remodelling, mRNA processing and transcription termination [28], [29], [30], [31]. For example, Ess1 has been shown to stimulate the dephosphorylation of Ser5 to efficiently terminate transcription of a subset of genes [24].

In this study, we analyzed how Rrd1 regulates transcription by RNAPII. We mapped Rrd1 and RNAPII occupancy using ChIP-chip analysis in the presence and the absence of rapamycin. We found that Rrd1 colocalized with RNAPII on actively transcribed genes under both conditions. Furthermore, *rrd1Δ* deletion affected RNAPII occupancy on a large set of rapamycin responsive genes. This was independent of TATA binding protein (TBP) recruitment to the promoter, suggesting that Rrd1 acts downstream of PIC formation during transcriptional initiation and elongation. The observation that Rrd1 modulated Ser5 and Ser2 phosphorylation of the RNAPII CTD further supported a role for Rrd1 in elongation. Finally, we demonstrate that Rrd1 is required to regulate gene expression in response to a variety of environmental stresses, thus establishing Rrd1 as a new elongation factor required for effective transcriptional responses to environmental challenges.

## Results

### Rrd1 affects RNAPII occupancy on rapamycin responsive genes

**Figure 1:**
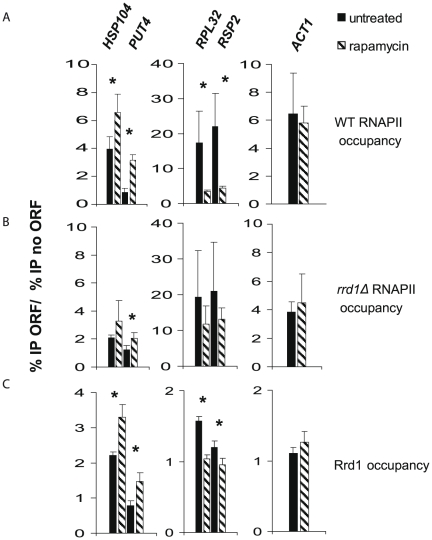
Rrd1 affects RNAPII occupancy on rapamycin responsive genes. (**A**) ChIP of Rpb1 (RNAPII) from WT cells followed by Q-PCR on the indicated genes. Dashed bars indicate rapamycin treatment (100 ng/ml for 30 min). The data is represented as a ratio of the % IP over Input on the ORF compared to % IP over Input in a non transcribed region (noORF). (**B**) ChIP of RNAPII from *rrd1Δ* mutant cells performed as in panel A. (**C**) ChIP of Rrd1-MYC (Myc antibodies) performed as in panel A. Results for all panels are shown as an average of at least three independent experiments, error bars indicate the standard deviation and the asterix (*) indicates if the *P*-value is below 0.05 between the untreated and treated condition using the Student T test.

Recently, we have shown that Rrd1 interacts with and isomerizes RNA polymerase II (RNAPII) in response to rapamycin [22]. Also it was demonstrated that Rrd1 is required to regulate the expression of some rapamycin responsive genes [20]. To further investigate this, we used ChIP analysis to measure the association of Rpb1, the major subunit of RNAPII (henceforth referred to as RNAPII), within the ORFs of four known rapamycin-responsive genes in wild-type cells and *rrd1Δ* mutant cells [32]. The rapamycin-upregulated genes, such as *HSP104* and *PUT4*, were significantly enriched for RNAPII in the wild-type strain, but this association was reduced in the *rrd1Δ* mutant (Fig. 1A and B). Examination of *RPL32* and *RPS2*, which are downregulated by rapamycin, revealed that RNAPII dissociated from both genes upon rapamycin treatment of wild-type yeast but remained bound in the *rrd1Δ* (Fig. 1A and B). Localization of RNAPII to *ACT1*, a gene unaffected by rapamycin treatment, was not altered upon addition of rapamycin in wild-type cells and or by *RRD1* deletion (Fig. 1A and B). These data indicate that Rrd1 is required to modulate expression of a larger set of genes than previously discovered [20].

To better understand by what mechanism Rrd1 affects transcription, we tagged Rrd1 with a Myc epitope and asked if Rrd1 also localizes to the set of genes assayed above. Similar to RNAPII, Rrd1 occupancy was increased on the ORFs of *HSP104* and *PUT4*, depleted on those of *RPL32* and *RPS2*, and remained constant on *ACT1*, in response to rapamycin (Fig. 1C). These data suggest that Rrd1 directly regulates these rapamycin-responsive genes.

### Rrd1 localization correlates with RNAPII along actively transcribed genes

**Figure 2:**
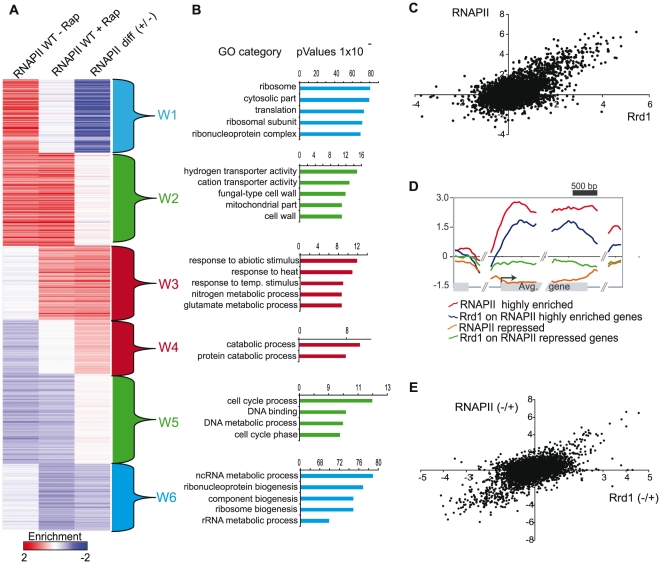
Rrd1 localization correlates with RNAPII along actively transcribed genes. (**A**) Self-organizing map (SOM) clustering analysis of the median enrichment of RNAPII on all ORFs from the WT strain. The red colour indicates enriched (bound) regions and blue colour represents depleted regions. The difference (diff (+/−)) (right column) was calculated by subtracting the complete untreated (−RAP) RNAPII data set (left column) from the treated (+RAP) RNAPII data set (middle row) (RAP = rapamycin 100 ng/ml for 30 min). The brackets (W1–W6) indicate clusters that were analyzed by gene ontology (GO). (**B**) Functional annotation of cluster W1–W6 with representative GO groups is shown. Funcassociate 2.0 was used to generate the full GO analysis, available in supplemental file S1. The X- axis represents the P-value. (**C**) Linear correlation of RNAPII and Rrd1-Myc enriched genes under normal growth condition. Each dot represents a single gene containing the median enrichment value from RNAPII on the y-axis and the Rrd1-Myc median enrichments on the x-axis. For the rapamycin treated data sets refer to supplemental figure S1A. (**D**) Mapping of RNAPII and Rrd1 on groups of genes with different median enrichment of RNAPII under normal growth conditions. The red line represents RNAPII along the 10% of genes with the highest amount of RNAPII and the blue line represents Rrd1 on this same group of genes. The orange line represents RNAPII on the lowest 10% RNAPII enriched genes and the green line represents Rrd1 on this same group of genes. (**E**) Linear correlation of RNAPII and Rrd1 gene occupancy differences between normal growth and rapamycin conditions. This is the same analysis as (C) only that the difference of the untreated and treated data set for the median enrichment of RNAPII and Rrd1 was plotted.

To extend our initial analysis from a small set of genes to the entire genome, we used the ChIP-chip assay to examine the genome-wide distribution of both RNAPII and Rrd1. We first examined RNAPII occupancy under exponential growth conditions and after a 30 minutes treatment with rapamycin, the time point at which the rapamycin transcriptional response is most prominent based on mRNA expression analysis [32]. ChIP samples were hybridized on tiling arrays covering the whole yeast genome with an average resolution of four probes per kilobase. We first calculated the median RNAPII occupancy over the coding region of approximately 5000 ORFs and clustered these enrichment values using self organizing maps (SOM) with *Cluster 3.0* and used Java TreeView to visualize the data [33]. Figure 2A shows the average RNAPII signal over each ORF in the absence (column 1), or the presence (column 2) of rapamycin. In order to better visualize the genes that were affected by rapamycin, we also show the difference in RNAPII occupancy between the two conditions (rapamycin treated data was subtracted from the untreated; column 3). We identified 6 clusters of genes (W1–W6) with different behaviors upon rapamycin treatment. These clusters were then analyzed for gene ontology (GO) category using *funcassociate 2.0* (Fig. 2B and Suppl. File S1) [34]. This analysis revealed that upon rapamycin treatment, RNAPII occupancy was sharply reduced on metabolic genes including those involved in ribosome biogenesis (see cluster W1, W6 and Fig. 2B). In contrast, RNAPII was strongly recruited to genes involved in nitrogen discrimination, the Krebs cycle, stress response and catabolic processes after rapamycin treatment (W3, W4 and Fig. 2B). These data are consistent with the transcriptional changes reported for rapamycin treatment as well as for environmental stress responses [32], [35].

We next performed ChIP-chip analysis on Myc-tagged Rrd1 and compared its distribution on the ORFs with that of RNAPII. As shown in Figure 2C, RNAPII occupancy correlates with that of Rrd1, indicating that Rrd1 is recruited to transcribed genes. Interestingly, when cells were treated with rapamycin, Rrd1 and RNAPII displayed a similar correlation (Suppl. Fig S1A), suggesting that under normal growth conditions, as well as after drastic transcriptional changes, Rrd1 remains associated to actively transcribed genes. To further investigate this relationship, we mapped RNAPII occupancy along a group of genes with the lowest levels of RNAPII as well as a group with the highest levels of RNAPII and then mapped Rrd1 on the same groups (Fig. 2D). Indeed, when RNAPII levels were low, the Rrd1 levels were also low, and consistently, when RNAPII levels were high Rrd1 levels were also increased (Fig. 2D). This was also the case for rapamycin treated cells (Suppl. Fig. S1B).

To further confirm that Rrd1 changes in occupancy correlate with RNAPII in response to rapamycin, we plotted the difference in RNAPII occupancy between untreated and rapamycin-treated cells versus the difference in Rrd1 occupancy between these two conditions (Fig. 2E). This analysis revealed that both, Rrd1 and RNAPII are downregulated on a large group of genes and recruited to another group of genes in response to rapamycin (R^2^ = 0.34). Taken together, this data suggest that Rrd1 and RNAPII co-localize on the coding region of most of the actively transcribed genes, even after massive transcriptional changes such as the ones caused by rapamycin treatment.

### *RRD1* is required for proper RNAPII relocalization in response to rapamycin

**Figure 3:**
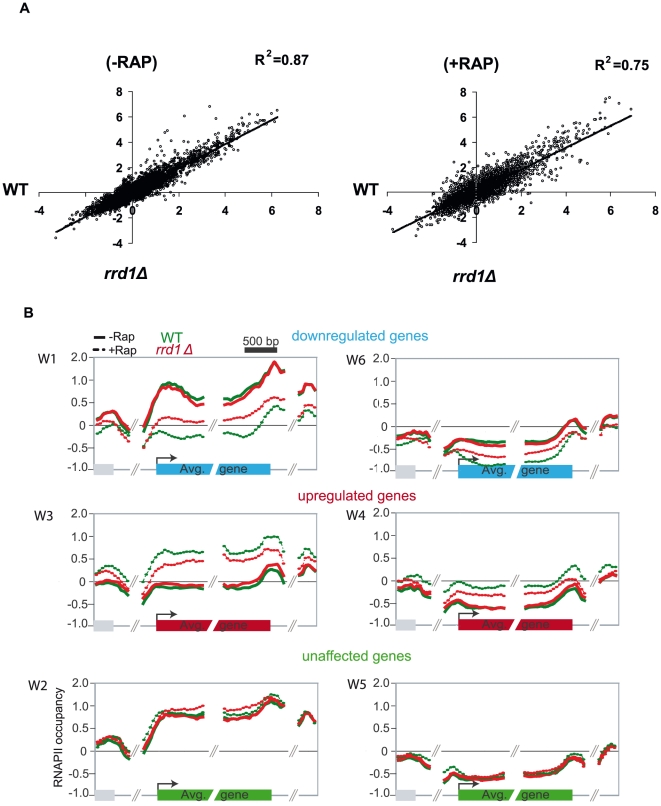
*rrd1Δ* deletion affects RNAPII localization in response to rapamycin. (**A**) Linear correlation of RNAPII median enrichment of WT (x-axis) and *rrd1Δ* mutant (y-axis) under normal growth conditions (−RAP) (left panel) and rapamycin treated conditions (+RAP) (right panel). The trend line and the R^2^ value are displayed for each condition. (**B**) Mapping of RNAPII from WT cells (green line) and *rrd1Δ* mutant cells (red line) under normal growth conditions (full line) and rapamycin treated conditions (dotted line) on cluster W1–W6 from figure 2A. Mappings are sorted for unaffected genes (green) upregulated genes (red) and downregulated genes (blue).

Since Rrd1 co-localizes with RNAPII on actively transcribed genes and Rrd1 was previously shown to be required to modulate the expression of a few rapamycin-responsive genes [20], we analyzed the genome-wide localization of RNAPII in the *rrd1Δ* mutant. To compare RNAPII levels from the wild-type and *rrd1Δ* cells, we plotted RNAPII occupancy in wild-type and *rrd1Δ* cells, both in absence and presence of rapamycin (Fig. 3A). Whereas RNAPII levels were similar (R^2^ = 0.87) in untreated cells, they were less correlated in response to rapamycin (R^2^ = 0.75), suggesting that Rrd1 is required to regulate RNAPII occupancy in response to rapamycin as observed at individual genes in figure 1. To further confirm this, we mapped RNAPII occupancy from wild-type and *rrd1Δ* mutant on the six clusters from Figure 2A (Fig. 3B). Interestingly, at down regulated genes, RNAPII in the *rrd1Δ* mutant failed to reach levels as low as in wild type cells (cluster W1 and W6). On the other hand, on upregulated genes (cluster W3 and W4) RNAPII also failed to reach wild-type levels in the *rrd1Δ* mutant. We note that RNAPII occupancy was similar for the untreated RNAPII levels (full line) as well as for clusters that were not affected by rapamycin (cluster W2 and W5). Taken together, this data clearly indicates that Rrd1 is required for the optimal up and down regulation of RNAPII levels during rapamycin response.

### Rrd1 regulates RNAPII occupancy independently of TBP binding

**Figure 4:**
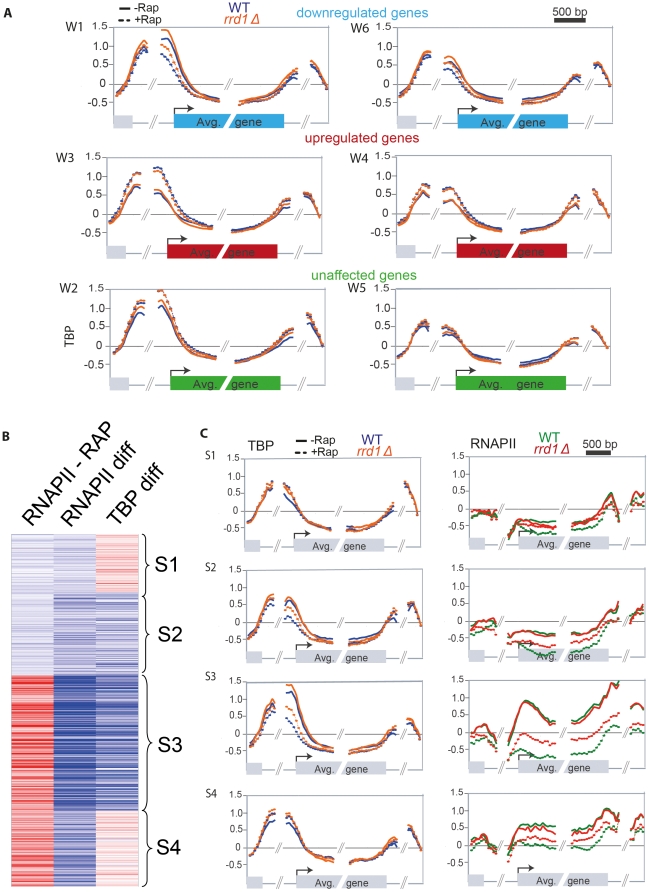
Rrd1 is required for RNAPII association independently of TBP recruitment. (**A**) Mapping of TBP-MYC occupancy on genes from clusters W1–W6, from WT (blue) and from *rrd1Δ* mutant (orange) under normal growth condition (full line) and rapamycin treatment (dotted line). (**B**) SOM clustering of cluster W1 and W6 of RNAPII under normal growth conditions (−RAP), the difference of RNAPII from treated and untreated conditions (diff) and the difference of TBP from treated and untreated conditions (diff). The red colour indicates enriched (bound) regions and blue colour represents depleted regions. (**C**) Mapping analysis of the clusters S1–S4 generated from (A) of TBP (left panel) and RNAPII (right panel). Legend is the same as (A) for TBP and the same as Fig. 3B for RNAPII.

To find out at what step Rrd1 influences RNAPII transcription, we examined the genome wide association of the yeast Myc-tagged TATA box binding protein (TBP) encoded by *SPT15* using ChIP-chip. If, upon rapamycin treatment, Rrd1 affects steps upstream of PIC formation, TBP occupancy would be affected in *rrd1Δ* cells. In the other scenario, whereby Rrd1 was to affect a step downstream from PIC formation (such as elongtion for example), TBP levels (contrarily to RNAPII levels, Figure 3B) would not necessarily be affected. We therefore mapped TBP occupancy in WT (blue) and *rrd1Δ* cells (orange) on genes from clusters W1–W6 (Fig. 4A). Interestingly, at upregulated genes (cluster W3 and W4) TBP binding (unlike RNAPII occupancy) is not affected by the deletion of *RRD1*. This shows that the upregulation of RNAPII is controlled by Rrd1 independently of TBP binding and suggest that Rrd1 may regulate transcriptional elongation as will be addressed below.

We next checked if the regulation of the rapamycin-downregulated genes by Rrd1 is TBP-independant. Preliminary analysis of the downregulated genes (clusters W1 and W6) suggested that these are heterogeneous with respect to TBP binding (Fig. 4A). We therefore generated new clusters from clusters W1 and W6 using both RNAPII and TBP occupancy values from wild type cells (Fig. 4B). This clustering revealed that although RNAPII is downregulated for all genes TBP occupancy is only reduced in cluster S2 and S3 in wild-type cells treated with rapamycin. This suggests that, in wild-type cells, rapamycin leads to gene expression downregulation by at least two distinct mechanisms. The first involving the regulation of TBP recruitment and the latter involving the regulation of a downstream step. To look at the function of Rrd1 in that regulation, we looked at TBP and RNAPII occupancies in *rrd1Δ* cells on these clusters (Figure 4C). As predicted from Figure 4A, deletion of *RRD1* affected RNAPII occupancy of all these genes upon rapamycin treatment. Interestingly, however, TBP levels were not affected by the deletion of *RRD1* for the genes included in clusters S1 and S4, while they were significantly crippled at genes from clusters S2 and S3. Taken together these data indicate that RNAPII downregulation upon rapamycin treatment is regulated by two mechanisms, one which does not depend on TBP depletion (cluster S1 and S4). Interestingly, Rrd1 is required for both mechanisms by optimizing TBP depletion (S2 and S3) and RNAPII levels (S1 and S4). This suggests that Rrd1 has an influence on TBP binding, probably by affecting the signaling cascade upstream of PIC assembly [19] but also functions downstream of TBP binding, likely during transcription initiation/elongation.

### Rrd1 functions during transcription elongation

**Figure 5:**
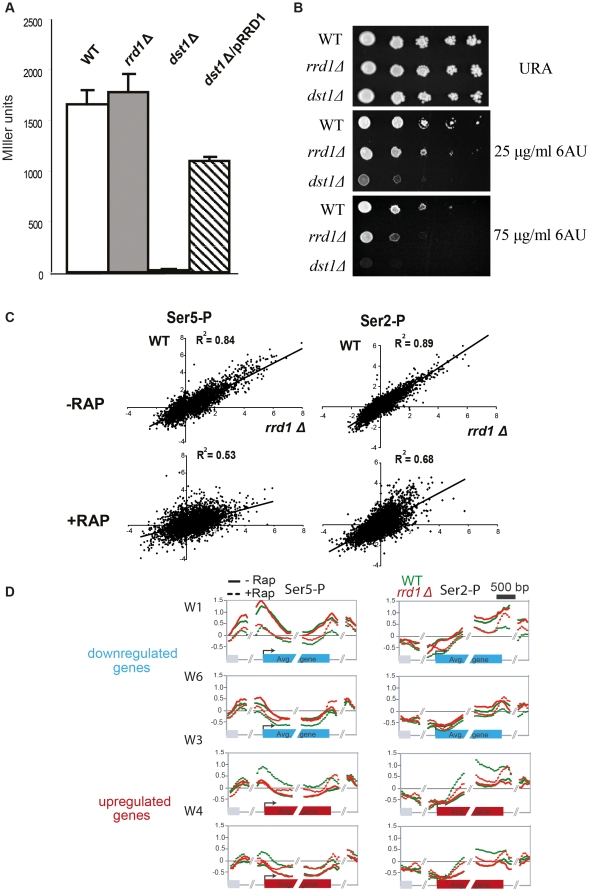
*rrd1Δ* mutants exhibit phenotypes consistent with a defect in transcriptional elongation. (**A**) Transcription elongation assay measuring the β-galactosidase activity of the *lacZ* gene containing the artificial arrest site expressed from a plasmid transformed into the indicated strains. The *dst1Δ* mutant strain was co-transformed with a plasmid expressing *RRD1* (pRRD1). The measurements are expressed as Miller units and are displayed as the average of at least 5 independent assays with the corresponding error bars. (**B**) Spot test analysis of WT, *rrd1Δ* and *dst1Δ* mutant strains on agar containing selective media lacking uracil (URA) and containing 6-azauracil (6AU) with the indicated concentration. (**C**) Linear correlations of the mean enrichment of Ser5 phosphorylated form of RNAPII (Ser5-P) at all promoters and Ser2 phosphorylated form of RNAPII (Ser2-P) of all ORFs, between WT and *rrd1Δ* mutant without (− RAP) and with (+ RAP) rapamycin treatment. (**D**) Mapping analysis of Ser5-P and Ser2-P forms of RNAPII for up and downregulated genes in response to rapamycin. WT cells (green line) and *rrd1Δ* mutant cells (red line) under normal growth conditions (full line) and rapamycin treated conditions (dotted line).

The above data suggest that Rrd1 might regulate RNAPII during transcription initiation and/or elongation. To test this possibility we used a well known transcription elongation assay that is based on an artificial arrest site (ARTAR) within the *E.coli* β- galactosidase ORF and is thought to cause RNAPII stalling/pausing downstream of the promoter during elongation [25], [36]. This ARTAR is composed of three arrest sites which promote backtracking and arresting of RNAPII and elongation factors such as Dst1 are required to restart RNAPII so that it can resume transcription [25], [36]. We introduced this reporter gene expression plasmid in the wild-type, *rrd1Δ* mutant and *dst1Δ* mutant strains and measured LacZ expression (Fig. 5A). Similar to wild-type, *rrd1Δ* mutants were able to overcome the ARTAR and express LacZ as opposed to the *dst1Δ* mutants, suggesting that Rrd1 is not essential to overcome RNAPII arrests in this system. We next tested whether increased dosage of Rrd1 would rescue the elongation defect of *dst1Δ* mutants (Fig. 5A). Strikingly, ectopic overexpression of Rrd1 partially restored the lacZ expression in the *dst1Δ* mutant. This clearly indicates that although Rrd1 is not essential for transcription elongation it is likely to perform a backup function during elongation or alternatively that Rrd1 promotes elongation of RNAPII in such a way that it overcomes the ARTAR.

Many mutants defective in transcription elongation, such as *dst1Δ* and *spt4Δ*, are known to be sensitive to 6-azauracil (6-AU), an inhibitor of the IMP dehydrogenase, which decreases GTP pools and thereby causes transcriptional arrest [36]. We found that *rrd1Δ* mutants were sensitive to 6-AU, although not as sensitive as *dst1Δ* cells (Fig. 5B), further supporting a role for Rrd1 in transcription elongation.

Finally, we examined whether Rrd1 might influence elongation by altering the phosphorylation status of the RNAPII C-terminal domain (CTD). The CTD is highly phosphorylated on Ser5 at the promoter and in the early elongation phase, and is progressively dephosphorylated as transcription progresses. In contrast, Ser2 phosphorylation gradually increases throughout the ORF, reaching maximum levels at the 3′ end of the gene [28], [29], [30], [31]. These differential phosphorylation states can be used to monitor transcription elongation efficiency [24], [27]. We used phosphospecific antibodies to perform ChIP-chip analysis for Ser5 phosphorylation (Ser5-P) and Ser2 phosphorylation (Ser2-P) in wild-type and *rrd1Δ* strains with and without rapamycin. Figure 5C shows the correlation between wild-type and *rrd1Δ* for Ser5-P and Ser2-P under untreated and rapamycin treated conditions. The correlation of Ser2-P shifted from a R^2^ = 0.89 to an R^2^ = 0.68 upon rapamycin treatment and the correlation of Ser5-P was even more affected upon treatment (R^2^ = 0.84 and R^2^ = 0.53). This suggests that Rrd1 influences the phosphorylation status of RNAPII in response to rapamycin. We next mapped Ser5-P and Ser2-P from wild-type and *rrd1Δ* mutant on clusters W1 to W6 (Fig. 5D and Suppl. Fig. S2A). We found that in general the pattern of Ser5-P and Ser2-P is similar between wild-type and *rrd1Δ* mutant under normal growth condition (full line). However, when treated with rapamycin (dashed line) the patterns of both phosphorylations were altered in the *rrd1Δ* mutant in each cluster, leading to an increase in the 3′ region of the genes. On down regulated genes, Ser5-P largely remained at the 3′end in the mutant as compared to wild-type for cluster W1 and increased for cluster W6 (Fig. 5D). In the case of the upregulated genes (W3 and W4), Ser5-P increased in the wild-type at promoters and within the ORF but not in the mutant where it remained low except for being elevated at the 3′ end of these genes. Similarly to Ser5-P, Ser2-P remained high at the 3′ end of genes in the mutant on down regulated genes (W1 and W6). Also for the upregulated genes, Ser2-P was lower in the mutant throughout the ORFs but then peaked only at the 3′ end as compared to wild-type (W3 and W4). We next analyzed Ser5-P and Ser2-P on the clusters S1 to S4 and here also, both phosphorylations were similarly altered in response to rapamycin (Suppl. Fig S2B). Taken together, this indicates that Rrd1 affects the phosphorylation states of RNAPII in response to a transcriptional stress (see discussion).

### Rrd1 plays a role in environmental stress responses

**Figure 6:**
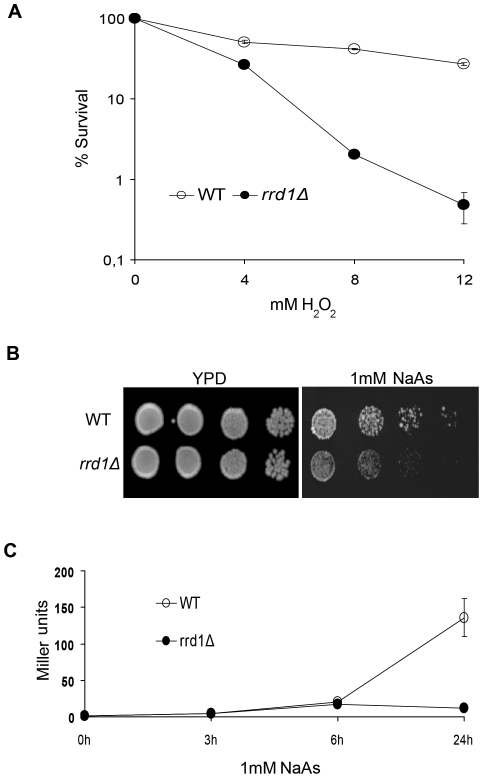
*rrd1Δ* mutants display hypersensitivity to agents causing oxidative stress. (**A**) Survival curve of WT (open circle) and *rrd1Δ* mutant strains (closed circle) upon H_2_O_2_ treatment. H_2_O_2_ concentrations are indicated below; result shown is an average of three independent experiments and error bars indicate the standard deviation. (**B**) Spot test analysis of WT and *rrd1Δ* mutant strain on YPD agar plates, containing 1 mM sodium arsenite (NaAs), where indicated. (**C**) LacZ reporter expression analysis from the *ACR3* promoter-lacZ fusion plasmid, expressed in Miller units. WT (open circle) and *rrd1Δ* mutant (closed circle) show the lacZ expression over time in response to 1 mM NaAs treatment (*n* = 5).

The above data indicate that Rrd1 is required for an optimal transcriptional response following rapamycin exposure. Since rapamycin mimics nutrient starvation conditions, we next asked whether Rrd1 is required for optimal response to other environmental stresses. Previously work has shown that *rrd1Δ* mutants exhibit multiple phenotypes including resistance to caffeine, but sensitivity towards vanadate, 4-NQO and calcium [13], [37]. Both vanadate and 4-NQO are known to cause oxidative stress [37], [38]. To test whether Rrd1 is required for resistance to other oxidizing agents, we challenged cells with hydrogen peroxide (H_2_O_2)_ and sodium arsenite (NaAs) and found that the *rrd1Δ* mutant was indeed sensitive to these agents (Fig. 6A and B). To determine whether the sensitivity was the result of a defect in gene regulation, we introduced a known arsenite-response reporter that bears the promoter of the *ACR3* gene fused to lacZ [39]. *ACR3* encodes a plasma membrane efflux pump that is upregulated *via* the Yap8 transcriptional activator in response to arsenite [39]. While there was a strong induction of the *ACR3*-lacZ reporter in the wild-type, it was hardly induced in the *rrd1Δ* mutant (Fig. 6C). This data suggests that the transcriptional response to oxidative stress is also affected in the *rrd1Δ* mutant.

**Figure 7:**
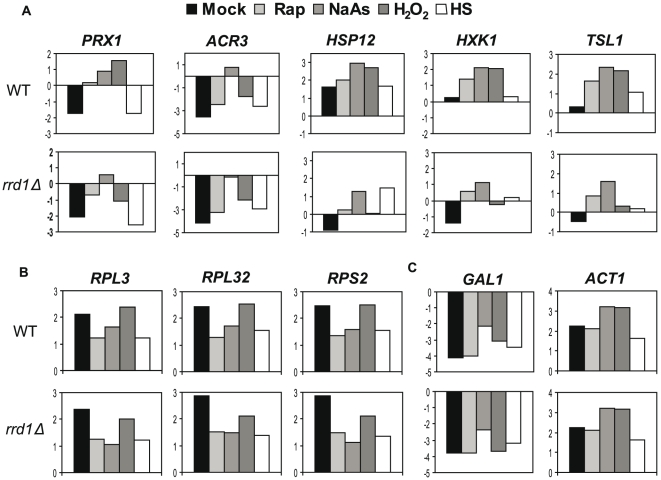
mRNA expression analyses in response to different stress conditions. GeXP multiplex expression analysis of 10 genes from untreated, rapamycin (100 ng/ml for 30 min), H_2_O_2_ (0.6 mM for 30 min), NaAs (1 mM for 30 min), heat shock (37°C for 30 min) treated conditions. The data is expressed in log 2 ratio and is calculated relative to an internal PCR control. (**A**) Five upregulated genes. (**B**) Three downregulated genes (**C**) Two control genes.

To explore this further, we monitored expression of 10 stress responsive genes using the GeXP multiplex PCR system in response to rapamycin, H_2_O_2_, NaAs and heat shock (see Materials and Methods). We chose genes that are known to be upregulated (*PRX1*, *ARR3*, *HSP12*, *HXK1* and *TSL1*) or downregulated (*RPL3*, *RPL32*, and *RPS2*) in response to environmental stresses as well as control genes which are not significantly altered under these conditions (*ACT1*, *GAL1*) [35]. The Kanamycin resistance marker was added to the samples and used to normalize the data (see materials and methods). First, the untreated and rapamycin-treated expression data was compared to the RNAPII median enrichment on these ORFs (see Suppl. Fig. S3). This analysis revealed that for both conditions RNAPII occupancy correlated with mRNA expression for all of the genes except the ribosomal genes (see Suppl. Fig. S3). This can be explained by the mRNA half life of the downregulated genes: Although RNAPII is depleted from these genes, mRNA is still present. We next compared gene expression in wild-type and *rrd1Δ* yeast for each condition (Fig. 7). Genes that are known to be induced were indeed upregulated in wild-type, but this was inhibited in the *rrd1Δ* mutant (Fig. 7A). Genes that are known to be downregulated upon stress were unaffected by *RRD1* deletion (Fig. 7B). It might be possible that for these genes other regulatory mechanisms are active such as the above mentioned mRNA half life (see discussion). This is consistent with the observation that RNAPII was strongly depleted from these genes in response to rapamycin, but mRNA levels remained unchanged (Suppl. Fig. S3). Finally, the control genes *ACT1* and *GAL1* remained similar between wild-type and *rrd1Δ* throughout the various conditions (Fig. 7C). Taken together, the expression analysis and the multiple phenotypes of *rrd1Δ* mutants towards environmental stresses suggest that the role of Rrd1 is not limited to rapamycin, but instead that this peptidyl-prolyl isomerase plays a general role in transcriptional stress responses.

## Discussion

We have previously shown that Rrd1 is associated to chromatin and interacts with RNAPII [22]. Consistently with this, we now demonstrate that Rrd1 directly co-localizes with RNAPII on actively transcribed genes independently of the massive transcriptional changes that are induced by rapamycin. Interestingly, our mapping studies revealed that Rrd1 association was restricted to the body of the ORFs, since it reached its highest levels downstream of the promoter as compared to RNAPII. In addition, at the 3′ gene ends Rrd1 occupancy was reduced before RNAPII. Numerous other elongation factors such as Dst1 (TFIIS) were also found to interact with RNAPII and to be associated within the body of the gene [40], [41]. These co-localisation events occurring only during elongation suggest a function of Rrd1 during this phase of transcription. This is further supported by the fact that *rrd1Δ* deletion affects RNAPII gene occupancy in response to rapamycin for up and down regulated genes. It is noteworthy that this effect is noticeable during elongation, as the alterations of RNAPII are visible within the body of the gene as opposed to accumulation at the promoter or 3′ end region. This implies that Rrd1 regulates the amount of transcribing RNAPII, which could be through at least two different mechanisms; one by recruiting more RNAPII to these genes or two, by regulating the speed by which RNAPII is transcribing along the genes.

Our analysis of TBP occupancy indicated that Rrd1 acts upstream as well as downstream of TBP association and thus might be involved in the recruitment as well as the regulation of RNAPII elongation (Figure 4). Upon rapamycin treatment, transcription factors translocate to the nucleus and are recruited to promoters to stimulate or repress transcription by regulating TBP and PIC assembly [4], [5], [8], [42], [43], [44]. How Rrd1 might operate upstream of TBP is unclear, although it could be through the ability of Rrd1 to activate phosphatases [17], [18], [45]. For example, Rrd1 could regulate a specific phosphatase thereby altering the recruitment of transcription factors and PIC assembly. We note that TBP remained promoter-bound on a subset of down regulated genes from which RNAPII dissociated in response to rapamycin. This was partially dependent on Rrd1 since RNAPII was less drastically reduced in the *rrd1Δ* mutant (Fig. 4C cluster S1 and S4). This could be a mechanism to temporarily downregulate transcription, while allowing for rapid restart of gene expression once the stress is over. That Rrd1 influences a larger number of genes downstream of PIC assembly suggests that this is the major role of Rrd1 in the transcriptional response to rapamycin. This, and given that Rrd1 is associated within the ORF of most genes, indicates that it acts at the level of elongation. Also, although Rrd1 is constantly associated with RNAPII, deletion of *RRD1* has only a tangible effect on RNAPII in response to stress, meaning that under normal growth conditions Rrd1 is not crucial for RNAPII recruitment to genes. There are several more evidences advocating that Rrd1 acts on RNAPII during initiation and/or elongation: **(i)** We have previously shown that Rrd1 is localized to the chromatin, interacts with RNAPII and directly isomerizes *in vivo* and *in vitro* it's CTD in response to rapamycin [22]. Many transcription elongation factors are known to interact with and modify the CTD, including Pin1, Fcp1 and the Bur kinases and the CTD is thought to be a recruitment platform for elongation, RNA processing and termination factors [28], [29], [30], [31]. **(ii)** Here we have shown that overexpression of *RRD1* suppresses the elongation defect of *DST1* (TFIIS) deletion in an artificial elongation assay (ARTAR). Dst1 is crucial to transcribe this ARTAR as it is required to restart paused RNAPII [25], [36]. Although we currently cannot explain how *RRD1* increased dosage rescues the *dst1Δ* deficiency, we presume that this might be *via* its function during elongation. **(iii)** *Rrd1Δ* mutants displayed hypersensitivity against the agent 6-AU, a phenotype which is common for elongation factors although it was not as sensitive as *dst1Δ* mutants, possibly because Rrd1 affects elongation at only a subset of genes whereas Dst1 acts globally [36], [40], [46], [47], [48], [49]. Finally, **(iv)** *rrd1Δ* mutants displayed an altered phosphorylation pattern for Ser5-P and Ser2-P on most genes (Fig. 5D and Suppl. Fig. S2). Phosphorylation of RNAPII changes throughout elongation and this pattern is altered in the *rrd1Δ* mutant. First of all, the phosphorylation pattern appears to be similar under normal growth conditions correlating with RNAPII levels. However, one observes a distinctive pattern of Ser5-P and Ser2-P in both up and downregulated genes in response to rapamycin. In the *rrd1Δ* mutant Ser5-P and Ser2-P are strongly enriched in the 3′ region of the genes consistently throughout all up and down regulated genes. So, how can the same phenomenon account for the failure to up and down regulate gene expression? This may be explained by the fact that for upregulated genes Ser5-P and Ser2-P is much higher in the WT throughout the ORF but not at the 3′ end of the gene compared to the *rrd1Δ* mutant. In this case, *rrd1Δ* fail to up regulate CTD phosphorylation and thus RNAPII levels do not increase. For down regulated genes *rrd1Δ* mutants retain the altered phosphorylation patterns at the 3′ end of the genes thereby prohibiting an adequate downregulation of RNAPII. Thus, Rrd1 would be required to modulate the phosphorylation of RNAPII so that they remain flexible for up and down regulation. If Rrd1 affects the phosphorylation status of RNAPII one would expect to see changes in the global phosphorylation status of RNAPII, for example analyzed by Western blot on total cell extract. However, we previously monitored the total phosphorylation status of RNAPII in response to rapamycin in the *rrd1Δ* mutant and could not find any significant differences [22]. This apparent discrepancy can be explained by the fact that the phosphorylation changes are very local (at the 3′ end of genes) and that for example, for the upregulated genes there is less phosphorylation in the *rrd1Δ* mutant in the ORF but then retains a higher level of phosphorylation in the 3′-end of downregulated genes. These subtle changes are therefore unlikely to be visible using the immunoblot approaches.

We propose a model in which Rrd1 regulates elongation by modulating the level of Ser5-P and Ser2-P *via* isomerisation of the CTD of RNAPII. The isomerization of the CTD of RNAPII would allow the efficient up and downregulation of RNAPII on stress regulated genes. Our model has some precedent, as another peptidyl-prolyl isomerase, Ess1, has been shown to regulate Ser5-P of RNAPII at the end of snRNAs genes, thereby promoting transcription termination via the Nrd1 pathway [24]. In addition, over expression of Pin1 (the human homolog of Ess1) results in hyper phosphorylation of RNAPII and its release from the chromatin [27]. It is known that RNAPII occupancy is regulated during transcription elongation, for example, it was previously reported that RNAPII was enriched on ribosomal genes but associated with a slow transcriptional rate [50]. Interestingly, when these cells were transferred from glucose to galactose containing medium, the level of RNAPII decreased on these ribosomal genes and their transcriptional rate increased. Simultaneously, RNAPII was recruited to other genes including those involved in mitochondrial function [50]. Similar to a switch from glucose to galactose, rapamycin induces a transcriptional response which requires some genes to be turned off and others to be induced. Rrd1 might promote this transcriptional reorganization by allowing Ser5-P and Ser2-P changes thereby fine-tuning the elongation efficiency.

Based on our model, we predicted that Rrd1 might play a similar role in other stress response situations, notably the environmental stress responses that induce a similar pattern of gene expression as rapamycin [35], [51]. Indeed, *rrd1Δ* mutants are sensitive to agents that cause oxidative stress, which is known to induce a drastic transcriptional response (Fig. 6) [35], [51]. Although these phenotypes may at first glance seem opposite of the one observed for rapamycin, they are actually consistent with our model of Rrd1 function: In both cases, the response to the stress condition is inhibited in *rrd1Δ* cells. This leads to resistance to rapamycin (because the environment remains nutrient-rich despite the presence of the drug), but sensitivity to oxidative stress (because the cells fail to arrest and adapt to high ROS conditions). In accordance with this, we show that Rrd1 is required to adequately induce gene expression on a subset of stress responsive genes upon various stress conditions (Fig. 7). Surprisingly, ribosomal genes were not strongly downregulated in wild-type cells as predicted from the ChIP-chip data. Since mRNA levels were measured at 30 min, long mRNA half-lives could obscure the drop in transcription that was apparent in the ChIP-chip data. Rrd1 was required for induction of stress-induced genes, however, consistent with our model. Interestingly, *rrd1Δ* cells showed stronger defects for some stresses than others. For example, *HSP12* induction was dependent on Rrd1 after rapamycin and H_2_O_2_, but not heat shock and to a lesser extend with NaAs (Fig. 7). This might be due to effects specific to each condition, as not all of the genes we tested were induced in the same manner by each condition. For example, the NaAs exporter *ACR3* was only expressed in response to NaAs, and this was dependent on Rrd1 despite the fact that expression was specific to one condition (Fig. 7).

Taken together, we have shown that Rrd1 regulates the transcriptional stress response *via* two mechanisms, through regulation of PIC assembly, and more drastically through regulation of RNAPII elongation. The latter mechanism is likely to be via CTD isomerisation and alteration of the phosphorylation status of RNAPII, thereby regulating the elongation of RNAPII. This is a general stress response mechanism since *rrd1Δ* mutants display multiple phenotypes and are unable to adequately regulate gene expression in response to environmental changes. As such, we propose that Rrd1 is a novel transcription elongation factor required to modulate the expression in response to environmental stresses. This study goes along with many others as it further supports that elongation is also subject to transcriptional regulation.

Finally, as Rrd1 is conserved throughout evolution, future work should examine whether this function is also remaining in higher eukaryotes as this could be a potential target for the TOR signalling pathway and cancer treatment.

## Materials and Methods

### Strains, cell growth and crosslinking conditions

All strains used in this study were from the BY4741 background (*Mat a*, *his3-1*, *leu2-0*, *met15-0*, *ura3-0*). The *RRD1* and *SPT15* genes were endogenously tagged as previously described [39]. For ChIP analysis cells were grown in 50 mL of YPD to an OD_600_ of 0.6–0.8 before crosslinking with 1% formaldehyde for 30 min.

### Chromatin Immunoprecipitation and Antibodies

ChIP experiments were performed as per [52], with minor modifications. For myc-tag ChIP, we used 5 µg of 9E11 antibody coupled to 2×10^7^ pan-mouse IgG DynaBeads (Invitrogen) per sample. RNAPII ChIPs were done using 2 µL of 8WG16 antibody coupled to 2×10^7^ panmouse IgG DynaBeads per sample. For Ser2-P and Ser5-P ChIP, we used 100 µL of rat serum (3E8 and 3E10 respectively) coupled to 2×10^7^ protein G DynaBeads per sample [53].

The microarrays used for location analysis were purchased from Agilent Technologies (Palo Alto, California, United States) and contain a total of 44,290 Tm-adjusted 60-mer probes covering the entire genome for an average density of one probe every 275 bp (±100 bp) within the probed regions (4X44K). Myc-tag ChIPs were hybridized against ChIPs from isogenic strains that did not contain the tag as controls. RNAPII ChIPs were hybridized against a sample derived from 400 ng of input (non-immunoprecipitated) DNA.

Quantitative real-time PCR analysis was performed using the ABI 7000 machine (Applied biosciences) and Sybr green PCR mastermix (Applied biosystems). The % IP ORF/% IP no ORF ratio was determined using the relative efficiency method and calculated as in Lloyd et al. [54]. Primers were designed using the primer express software (Applied biosciences) exclusively matching the ORF of the indicated genes (listed in table S1).

### Data Analysis

The data was normalized and replicates were combined using a weighted average method as described previously [52]. The log 2 ratio of each spot of combined datasets was then converted to Z-score, similar to Hogan et al. [55] to circumvent the large differences in the immunoprecipitation efficiencies of the different factors. Visual inspection of the Z-scores was carried out on the UCSC Genome Browser (http://genome.ucsc.edu/). All data analyses described here were done using data from protein-coding genes longer than 500 bp. Median Z-score values for promoter and gene coding sequences were calculated and used in our clustering analyses. Promoters are defined as the shortest of either 250 bp or half the intergenic region (half-IG) relative to the reference gene's 5′ boundary. Self-organizing map (SOM) clustering was done with the Cluster software [33] and visualized with Java Treeview [56]. Only genes with no missing value were used for clustering. Gene mapping was performed as in Rufiange et al. [57] on selected groups of genes described in the text. Briefly, data were mapped onto the 5′ and 3′ boundaries in 50 bp windows for each half-gene and adjacent half-IG regions. A sliding window of 300 bp was then applied to the Z-scores to smooth the curve.

GO analyses on clusters were performed with *funcassociate 2.0*. For this, an association file for the entire gene set was generated and used for all analyses [34].

Regression analysis was plotted using excel, where the x-axis and y-axis contained the whole data set of the average ORF enrichments of the indicated ChIP. A trend line was plotted and the R^2^ was calculated.

### Phenotype analysis of *rrd1Δ* mutants

The H_2_O_2_ survival curves were performed as described previously [58], briefly exponentially growing cells were washed once in 50 mM KPO4 (p H7.5) and treated with the indicated concentration of H_2_O_2_ (Bio basic Inc) in 50 mM KPO4 (pH 7.5) buffer for one hour. Cells were then scored for colony formation after three days at 30 C on YPD agar.

Spottest with NaAs and 6-azauracil (Sigma) analysis was performed as described previously [59]. For 6-azauracil strains were transformed with and empty vector bearing the URA3 gene (pTW423) and spotted onto synthetic media agar plates lacking uracil. For lacZ expression the plasmid bearing the *ACR3-*LacZ fusion was transformed into strains and exponentially growing cells were treated with 1 mM NaAs (Sigma), aliquots were taken and the β-gal assay was performed as described in [39], [60].

### mRNA extraction and GeXP analysis

Overnight cultures were sub-cultured and grown to an OD_600_ of 0.6–0.8. Cells were then treated with the indicated drugs for 30 minutes (0.6 mM H_2_O_2_, 1 mM NaAs and 100 ng/ml rapamycin). For heat shock, cells were spun down and resuspended in preheated media at 37°C for 30 min. mRNA extraction was performed with the RNeasy extraction kit (Qiagen), following user guide lines.

### Multiplex analysis by GeXP system

Primer design of the set of 10 genes (see suppl. Table S1) was done using the eXpress Designer module of the express Profiler software (Beckman Coulter). 25 ng RNA were used in a 20 µL reaction volume for RT. Kanamycin RNA, an internal positive control was included. The RT reaction was performed using the GenomeLab ™ GeXP Start Kit (Beckman Coulter) under the conditions: 1 min at 48°C, 60 min at 42°C, 5 min at 95°C, hold at 4°C, in a thermal cycler. After the RT, a PCR was performed under the conditions: 10 min at 95°C, followed by 35 cycles of 30 s at 94°C, 30 s at 55°C, and 1 min at 70°C. Expression analysis was carried out with the GenomeLab ™ GeXP system (Beckman-Coulter) following manufacturer's instructions using the fragment analysis method. The data was normalized to Kanamycin before being expressed as a log 2 ratio.

### Statistical analysis

For Q-PCR, survival curves and lacZ expression analysis at least three independent experiments were performed and the standard deviation and *P*-values were calculated (Student's T-test).

For GeXP analysis the percentage CV was calculated for each replicate. Only % CV ≤20% was taken for analysis. Values>2 SD were excluded before final calculation.

## Supporting Information

Table S1 A list of all primers used for the GeXP and Q-PCR analysis.
(TIF)

File S1 GO analysis of figures 2A.
(XLS)

Figure S1 (**A**) A complement to Figure 2C, same experiment besides that the data was obtained from rapamycin treated cells. (**B**) A complement of Fig. 2D, the same experiment besides that the data was obtained from rapamycin treated cells.
(TIF)

Figure S2 (**A**) Contains the mapping analysis of Ser5-P and Ser2-P of rapamycin unaffected genes (cluster W2 and W5). (**B**) Mapping of Ser5-P and Ser2-P on clusters S1–S4 from figure 4B.
(TIF)

Figure S3 Comparison of GeXP mRNA expression and RNAPII median average ChIP data of the corresponding gene for untreated and rapamycin treated conditions. Both are expressed in log2 ratio.
(TIF)

## References

[1] Dowling RJ, Pollak M, Sonenberg N (2009). Current status and challenges associated with targeting mTOR for cancer therapy.. Bio Drugs.

[2] De Virgilio C, Loewith R (2006). The TOR signalling network from yeast to man.. Int J Biochem Cell Biol.

[3] Wullschleger S, Loewith R, Hall MN (2006). TOR signaling in growth and metabolism.. Cell.

[4] Martin DE, Soulard A, Hall MN (2004). TOR regulates ribosomal protein gene expression via PKA and the Forkhead transcription factor FHL1.. Cell.

[5] Duvel K, Santhanam A, Garrett S, Schneper L, Broach JR (2003). Multiple roles of Tap42 in mediating rapamycin-induced transcriptional changes in yeast.. Mol Cell.

[6] Jacinto E, Guo B, Arndt KT, Schmelzle T, Hall MN (2001). TIP41 interacts with TAP42 and negatively regulates the TOR signaling pathway.. Mol Cell.

[7] Jacinto E, Hall MN (2003). Tor signalling in bugs, brain and brawn.. Nat Rev Mol Cell Biol.

[8] Uffenbeck SR, Krebs JE (2006). The role of chromatin structure in regulating stress-induced transcription in Saccharomyces cerevisiae.. Biochem Cell Biol.

[9] Huisinga KL, Pugh BF (2004). A genome-wide housekeeping role for TFIID and a highly regulated stress-related role for SAGA in Saccharomyces cerevisiae.. Mol Cell.

[10] Chan TF, Carvalho J, Riles L, Zheng XF (2000). A chemical genomics approach toward understanding the global functions of the target of rapamycin protein (TOR).. Proc Natl Acad Sci U S A.

[11] Neklesa TK, Davis RW (2008). Superoxide anions regulate TORC1 and its ability to bind Fpr1:rapamycin complex.. Proc Natl Acad Sci U S A.

[12] Xie MW, Jin F, Hwang H, Hwang S, Anand V (2005). Insights into TOR function and rapamycin response: chemical genomic profiling by using a high-density cell array method.. Proc Natl Acad Sci U S A.

[13] Rempola B, Kaniak A, Migdalski A, Rytka J, Slonimski PP (2000). Functional analysis of RRD1 (YIL153w) and RRD2 (YPL152w), which encode two putative activators of the phosphotyrosyl phosphatase activity of PP2A in Saccharomyces cerevisiae.. Mol Gen Genet.

[14] Jordens J, Janssens V, Longin S, Stevens I, Martens E (2006). The Protein Phosphatase 2A Phosphatase Activator Is a Novel Peptidyl-Prolyl cis/trans-Isomerase.. J Biol Chem.

[15] Cayla X, Goris J, Hermann J, Hendrix P, Ozon R (1990). Isolation and characterization of a tyrosyl phosphatase activator from rabbit skeletal muscle and Xenopus laevis oocytes.. Biochemistry.

[16] Cayla X, Van Hoof C, Bosch M, Waelkens E, Vandekerckhove J (1994). Molecular cloning, expression, and characterization of PTPA, a protein that activates the tyrosyl phosphatase activity of protein phosphatase 2A.. J Biol Chem.

[17] Fellner T, Lackner DH, Hombauer H, Piribauer P, Mudrak I (2003). A novel and essential mechanism determining specificity and activity of protein phosphatase 2A (PP2A) in vivo.. Genes Dev.

[18] Hombauer H, Weismann D, Mudrak I, Stanzel C, Fellner T (2007). Generation of active protein phosphatase 2A is coupled to holoenzyme assembly.. PLoS Biol.

[19] Zheng Y, Jiang Y (2005). The yeast phosphotyrosyl phosphatase activator is part of the Tap42-phosphatase complexes.. Mol Biol Cell.

[20] Douville J, David J, Lemieux KM, Gaudreau L, Ramotar D (2006). The Saccharomyces cerevisiae phosphatase activator RRD1 is required to modulate gene expression in response to rapamycin exposure.. Genetics.

[21] Lempiainen H, Shore D (2009). Growth control and ribosome biogenesis.. Curr Opin Cell Biol.

[22] Jouvet N, Poschmann J, Douville J, Bulet L, Ramotar D Rrd1 isomerizes RNA polymerase II in response to rapamycin.. BMC Mol Biol.

[23] Kops O, Zhou XZ, Lu KP (2002). Pin1 modulates the dephosphorylation of the RNA polymerase II C-terminal domain by yeast Fcp1.. FEBS Lett.

[24] Singh N, Ma Z, Gemmill T, Wu X, Defiglio H (2009). The Ess1 prolyl isomerase is required for transcription termination of small noncoding RNAs via the Nrd1 pathway.. Mol Cell.

[25] Wu X, Rossettini A, Hanes SD (2003). The ESS1 prolyl isomerase and its suppressor BYE1 interact with RNA pol II to inhibit transcription elongation in Saccharomyces cerevisiae.. Genetics.

[26] Xu YX, Hirose Y, Zhou XZ, Lu KP, Manley JL (2003). Pin1 modulates the structure and function of human RNA polymerase II.. Genes Dev.

[27] Xu YX, Manley JL (2007). Pin1 modulates RNA polymerase II activity during the transcription cycle.. Genes Dev.

[28] Buratowski S (2003). The CTD code.. Nat Struct Biol.

[29] Phatnani HP, Greenleaf AL (2006). Phosphorylation and functions of the RNA polymerase II CTD.. Genes Dev.

[30] Cho EJ (2007). RNA polymerase II carboxy-terminal domain with multiple connections.. Exp Mol Med.

[31] Egloff S, Murphy S (2008). Cracking the RNA polymerase II CTD code.. Trends Genet.

[32] Hardwick JS, Kuruvilla FG, Tong JK, Shamji AF, Schreiber SL (1999). Rapamycin-modulated transcription defines the subset of nutrient-sensitive signaling pathways directly controlled by the Tor proteins.. Proc Natl Acad Sci U S A.

[33] Eisen MB, Spellman PT, Brown PO, Botstein D (1998). Cluster analysis and display of genome-wide expression patterns.. Proc Natl Acad Sci U S A.

[34] Berriz GF, Beaver JE, Cenik C, Tasan M, Roth FP (2009). Next generation software for functional trend analysis.. Bioinformatics.

[35] Gasch AP, Spellman PT, Kao CM, Carmel-Harel O, Eisen MB (2000). Genomic expression programs in the response of yeast cells to environmental changes.. Mol Biol Cell.

[36] Kulish D, Struhl K (2001). TFIIS enhances transcriptional elongation through an artificial arrest site in vivo.. Mol Cell Biol.

[37] Ramotar D, Belanger E, Brodeur I, Masson JY, Drobetsky EA (1998). A yeast homologue of the human phosphotyrosyl phosphatase activator PTPA is implicated in protection against oxidative DNA damage induced by the model carcinogen 4-nitroquinoline 1-oxide.. J Biol Chem.

[38] Aureliano M, Crans DC (2009). Decavanadate (V10 O28 6-) and oxovanadates: oxometalates with many biological activities.. J Inorg Biochem.

[39] Wysocki R, Fortier PK, Maciaszczyk E, Thorsen M, Leduc A (2004). Transcriptional activation of metalloid tolerance genes in Saccharomyces cerevisiae requires the AP-1-like proteins Yap1p and Yap8p.. Mol Biol Cell.

[40] Ghavi-Helm Y, Michaut M, Acker J, Aude JC, Thuriaux P (2008). Genome-wide location analysis reveals a role of TFIIS in RNA polymerase III transcription.. Genes Dev.

[41] Cheung AC, Cramer P Structural basis of RNA polymerase II backtracking, arrest and reactivation.. Nature.

[42] Rohde JR, Bastidas R, Puria R, Cardenas ME (2008). Nutritional control via Tor signaling in Saccharomyces cerevisiae.. Curr Opin Microbiol.

[43] Schawalder SB, Kabani M, Howald I, Choudhury U, Werner M (2004). Growth-regulated recruitment of the essential yeast ribosomal protein gene activator Ifh1.. Nature.

[44] Wade JT, Hall DB, Struhl K (2004). The transcription factor Ifh1 is a key regulator of yeast ribosomal protein genes.. Nature.

[45] Longin S, Jordens J, Martens E, Stevens I, Janssens V (2004). An inactive protein phosphatase 2A population is associated with methylesterase and can be re-activated by the phosphotyrosyl phosphatase activator.. Biochem J.

[46] Riles L, Shaw RJ, Johnston M, Reines D (2004). Large-scale screening of yeast mutants for sensitivity to the IMP dehydrogenase inhibitor 6-azauracil.. Yeast.

[47] Exinger F, Lacroute F (1992). 6-Azauracil inhibition of GTP biosynthesis in Saccharomyces cerevisiae.. Curr Genet.

[48] Shaw RJ, Reines D (2000). Saccharomyces cerevisiae transcription elongation mutants are defective in PUR5 induction in response to nucleotide depletion.. Mol Cell Biol.

[49] Morillon A, Karabetsou N, O'Sullivan J, Kent N, Proudfoot N (2003). Isw1 chromatin remodeling ATPase coordinates transcription elongation and termination by RNA polymerase II.. Cell.

[50] Pelechano V, Jimeno-Gonzalez S, Rodriguez-Gil A, Garcia-Martinez J, Perez-Ortin JE (2009). Regulon-specific control of transcription elongation across the yeast genome.. PLoS Genet.

[51] Gasch AP (2007). Comparative genomics of the environmental stress response in ascomycete fungi.. Yeast.

[52] Ren B, Robert F, Wyrick JJ, Aparicio O, Jennings EG (2000). Genome-wide location and function of DNA binding proteins.. Science.

[53] Chapman RD, Heidemann M, Albert TK, Mailhammer R, Flatley A (2007). Transcribing RNA polymerase II is phosphorylated at CTD residue serine-7.. Science.

[54] Lloyd A, Pratt K, Siebrasse E, Moran MD, Duina AA (2009). Uncoupling of the patterns of chromatin association of different transcription elongation factors by a histone H3 mutant in Saccharomyces cerevisiae.. Eukaryot Cell.

[55] Hogan GJ, Lee CK, Lieb JD (2006). Cell cycle-specified fluctuation of nucleosome occupancy at gene promoters.. PLoS Genet.

[56] Saldanha AJ (2004). Java Treeview–extensible visualization of microarray data.. Bioinformatics.

[57] Rufiange A, Jacques PE, Bhat W, Robert F, Nourani A (2007). Genome-wide replication-independent histone H3 exchange occurs predominantly at promoters and implicates H3 K56 acetylation and Asf1.. Mol Cell.

[58] Vance JR, Wilson TE (2001). Repair of DNA strand breaks by the overlapping functions of lesion-specific and non-lesion-specific DNA 3′ phosphatases.. Mol Cell Biol.

[59] Aouida M, Page N, Leduc A, Peter M, Ramotar D (2004). A Genome-Wide Screen in Saccharomyces cerevisiae Reveals Altered Transport As a Mechanism of Resistance to the Anticancer Drug Bleomycin.. Cancer Res.

[60] Ralser M, Goehler H, Wanker EE, Lehrach H, Krobitsch S (2005). Generation of a yeast two-hybrid strain suitable for competitive protein binding analysis.. Biotechniques.

