# Supplementary figures and images for: The Peptidyl Prolyl Isomerase Rrd1 Regulates the Elongation of RNA Polymerase II during Transcriptional Stresses
Source: PLoS One. 2011 Aug 24;6(8):e23159. doi: 10.1371/journal.pone.0023159 (PMC3160861; doi:10.1371/journal.pone.0023159)

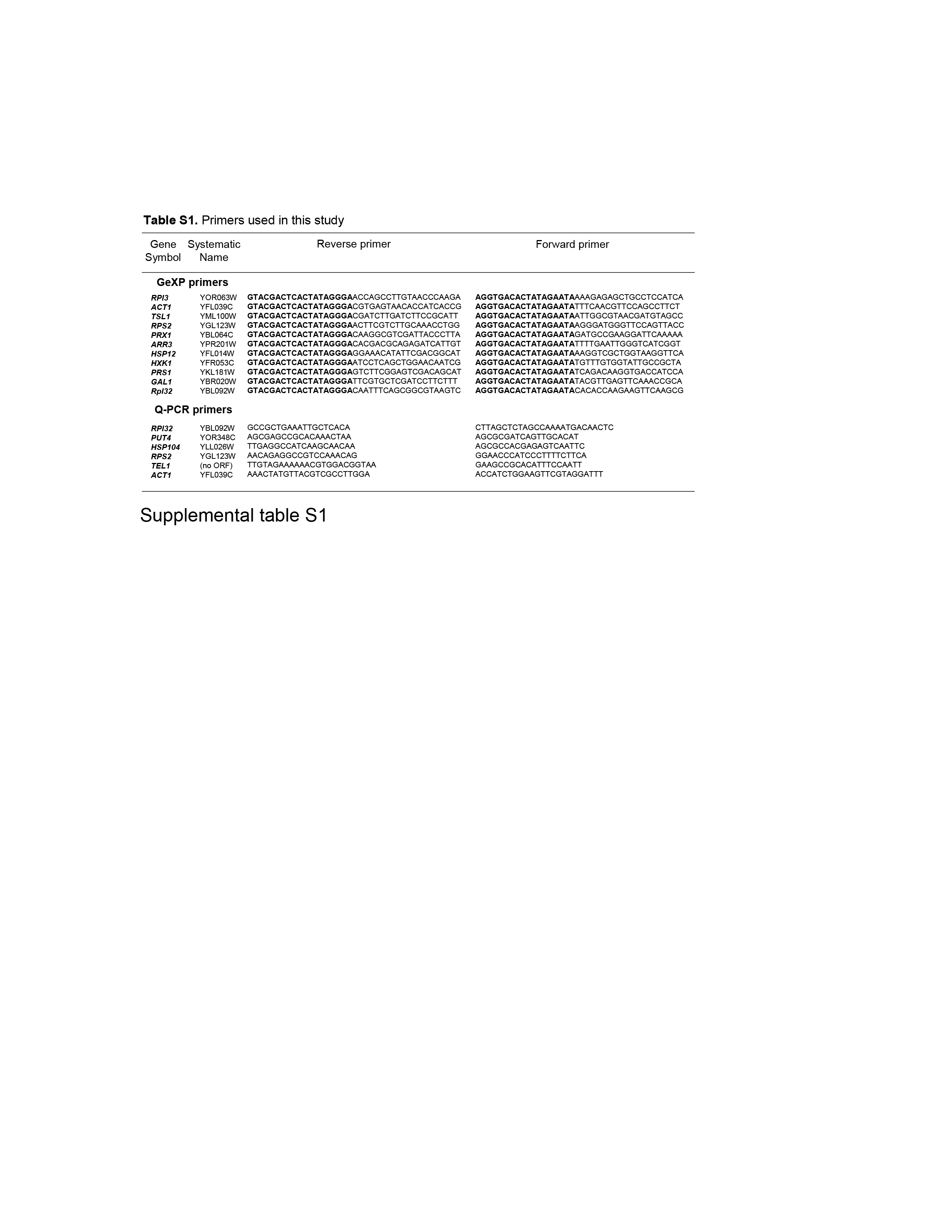

Supplement: Table S1 — A list of all primers used for the GeXP and Q-PCR analysis. (TIF) [file pone.0023159.s001.tif]

**A**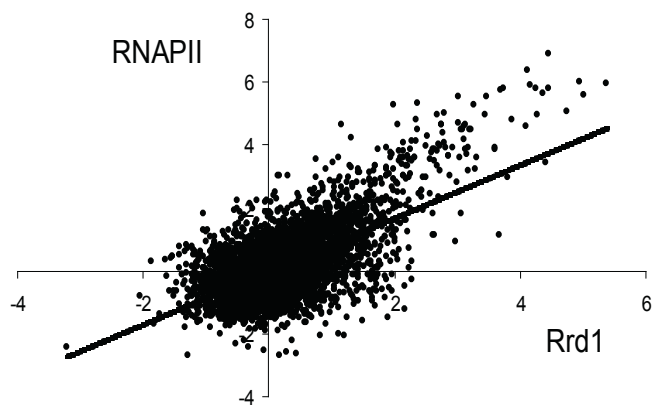**B**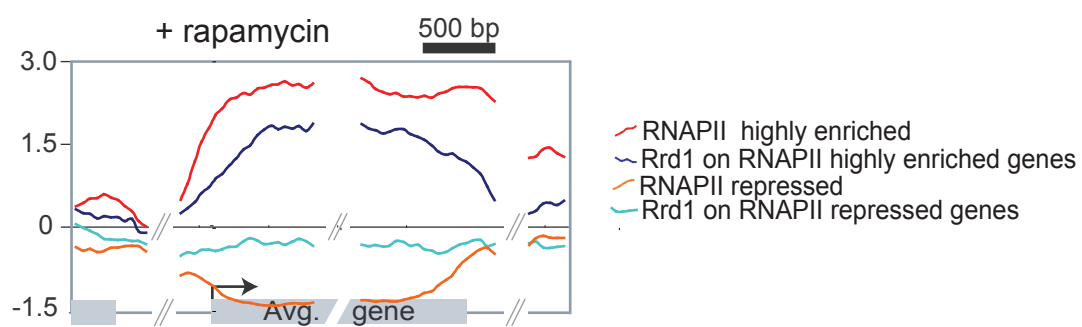

Supplemental Figure S1

Supplement: Figure S1 — (A) A complement to Figure 2C, same experiment besides that the data was obtained from rapamycin treated cells. (B) A complement of Fig. 2D, the same experiment besides that the data was obtained from rapamycin treated cells. (TIF) [file pone.0023159.s003.tif]

A

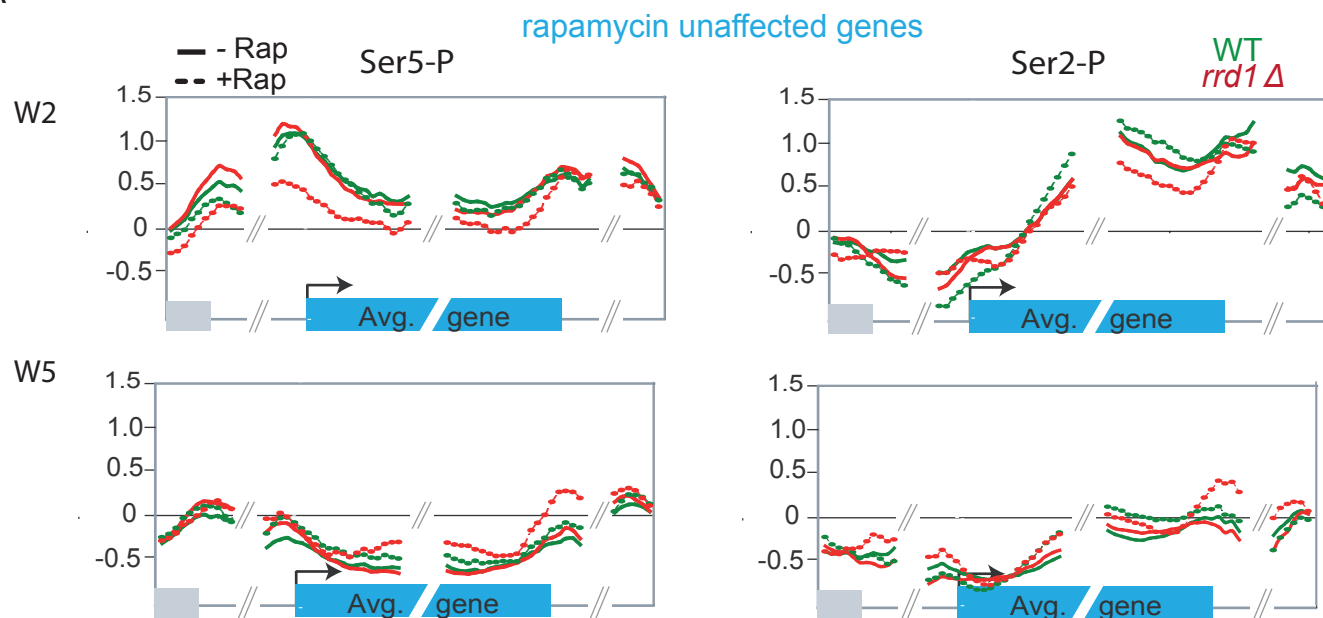

B

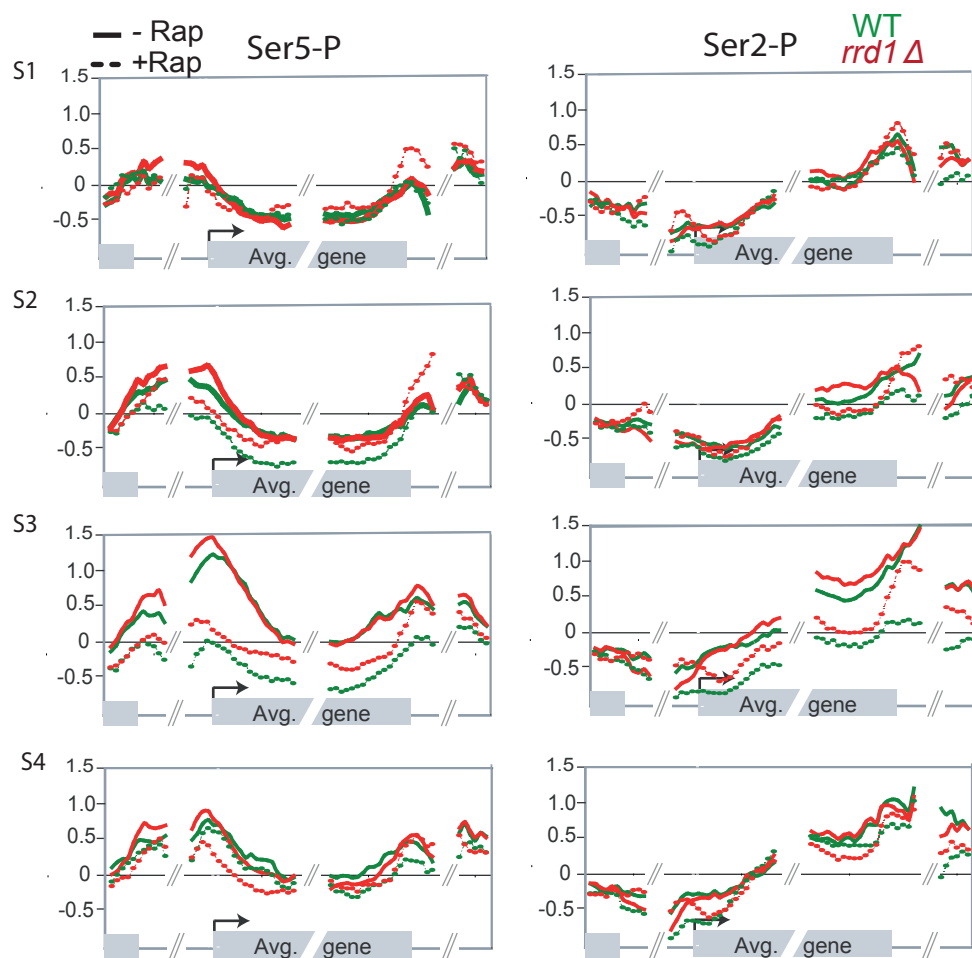

Supplemental Figure S2

Supplement: Figure S2 — (A) Contains the mapping analysis of Ser5-P and Ser2-P of rapamycin unaffected genes (cluster W2 and W5). (B) Mapping of Ser5-P and Ser2-P on clusters S1–S4 from figure 4B. (TIF) [file pone.0023159.s004.tif]

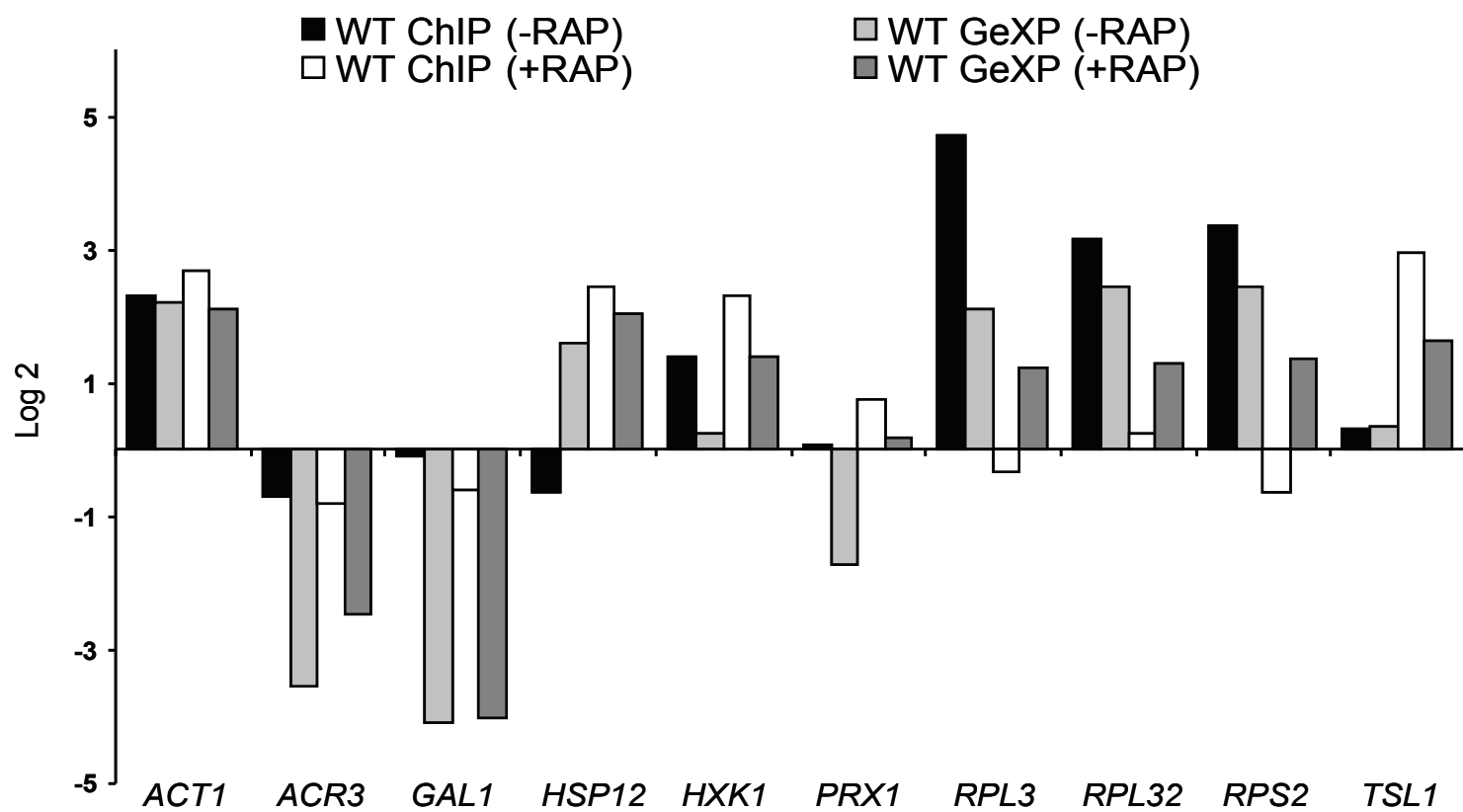

Supplemental Figure S3

Supplement: Figure S3 — Comparison of GeXP mRNA expression and RNAPII median average ChIP data of the corresponding gene for untreated and rapamycin treated conditions. Both are expressed in log2 ratio. (TIF) [file pone.0023159.s005.tif]
